# Target Dysbiosis of Gut Microbes as a Future Therapeutic Manipulation in Alzheimer’s Disease

**DOI:** 10.3389/fnagi.2020.544235

**Published:** 2020-10-06

**Authors:** Feiqi Zhu, Chunrong Li, Fengna Chu, Xiaoping Tian, Jie Zhu

**Affiliations:** ^1^Cognitive Impairment Ward of Neurology Department, The Third Affiliated Hospital of Shenzhen University Medical College, Shenzhen, China; ^2^Department of Neurology, Neuroscience Center, The First Hospital of Jilin University, Changchun, China; ^3^Division of Neurogeriatrcs, Department of Neurobiology, Care Sciences and Society, Karolinska Institutet, Karolinska University Hospital Solna, Stockholm, Sweden

**Keywords:** Alzheimer’s disease, gut microbiota, dysbiosis, microbiota-gut-brain axis, treatment

## Abstract

Alzheimer’s disease (AD) is commonly an age-associated dementia with neurodegeneration. The pathogenesis of AD is complex and still remains unclear. The inflammation, amyloid β (Aβ), and neurofibrillary tangles as well misfolded tau protein in the brain may contribute to the occurrence and development of AD. Compared with tau protein, Aβ is less toxic. So far, all efforts made in the treatments of AD with targeting these pathogenic factors were unsuccessful over the past decades. Recently, many studies demonstrated that changes of the intestinal environment and gut microbiota *via* gut–brain axis pathway can cause neurological disorders, such as AD, which may be involved in the pathogenesis of AD. Thus, remodeling the gut microbiota by various ways to maintain their balance might be a novel therapeutic strategy for AD. In the review article, we analyzed the characteristics of gut microbiota and its dysbiosis in AD and its animal models and investigated the possibility of targeting the gut microbiota in the treatment of the patients with AD in the future.

## Highlights

- The gut microbes communicate with the brain by several regulating pathways *via* the gut–brain axis involved in the physiological activities to maintain homeostasis of the human body. The imbalance of gut microbiota is associated with AD.
- The gut dysbiosis caused by several factors may aggravate neuroinflammation and other pathologies promoting the development and progression of AD.
- Targeting the gut dysbiosis or remodeling the gut microbiota might be a novel strategy for AD therapy.

## Introduction

Alzheimer’s disease (AD) is a chronic progressive neurodegenerative disorder and the most common form of age-associated dementia. In year 2017, it has been reported that about 40 million people suffered from AD in the world (Alzheimer’s Association, 2017; Esquerda-Canals et al., 2017). Despite a lot of previous intensive studies, the pathogenesis of AD remains insufficiently understood. Pathologic changes in the brain of AD include amyloid β (Aβ) plaque deposits and neurofibrillary tangles formed by intracellular accumulation of hyperphosphorylated tau protein and neuroinflammation. The pathological characteristics are current major theory of the pathogenesis of AD (Angelucci et al., 2019). However, the great efforts in therapeutic AD basis on the pathogenesis of AD with pathogenic Aβ or tau over the past decades have witnessed continuous failure, indicating that the pathogenesis of AD should be multifactorial and is more complex than a simple pathogenic Aβ or tau would suggest. With aging of human beings, the incidence of AD is rising continuously in the world, which has become a major public health problem (Angelucci et al., 2019). In order to develop the effective treatment, we need further a better understanding of the pathogenesis of AD.

Over the past 10 years, the researchers have been very concerned and interested in the role of the gut microbiome in modulating brain function, although the results were obtained mainly from animal models (Long-Smith et al., 2020). Microbiota may be a crucial predisposing factor for AD and other neurological disorders, which has been proven by a growing number of studies (Zhuang et al., 2018; Sochocka et al., 2019; Cryan et al., 2020; Long-Smith et al., 2020). AD has been considered as a systemic disease related to inflammation, and the inflammatory–infectious hypothesis of its pathogenesis becomes more significant (Bronzuoli et al., 2016). It has been evidenced that microbes and their products from the periphery infiltrating into the brain causing chronic inflammation are an important predisposing factor of neuroinflammation and neurodegenerative changes observed in AD (Cattaneo et al., 2017; Ashraf et al., 2019). AD and cognitive decline, as well as other neurodegenerative diseases, are often associated with gastrointestinal (GI) dysfunction (Zhuang et al., 2018; Sochocka et al., 2019; Ticinesi et al., 2019). It is postulated that AD may begin in the gut and is related to the imbalance of gut microbiota, while the intestinal inflammation and infections caused by various pathogens may control the changes of the gut microbiota first, and then other factors, as described below, are also involved in controlling these changes.

Throughout the course of these diseases, the GI disturbances may occur in the different stage of the diseases as a clinical manifestation. The alteration of enteric neuroimmune system (ENIS) and dysbiosis of the gut microbiota may lead to the occurrence of GI dysfunction and neurologic disorders (Pellegrini et al., 2018; Sochocka et al., 2019). Therefore, it has been hypothesized that AD is closely related to gut microbial alteration, and it is consistent with the pivotal role of inflammation in the pathogenesis of AD (Calsolaro and Edison, 2016; Haran et al., 2019). In the review article, we clarify the characteristics of the gut microbiota, analyze the role of dysbiosis of the gut microbes in the pathogenesis of AD, and discuss the possibility whether targeting the dysbiosis of gut microbes can be as a future therapeutic manipulation in AD.

## The Gut Microbiome and Their Role

### Features of the Gut Microbiota

Normal microorganisms in human being consist of bacteria, fungi, viruses, etc. and 95% of them are located in the large intestine (Swidsinski et al., 2005; Galland, 2014). The microbiota refers to bacteria, fungi, viruses, etc., existing in an ecosystem/habitat, and the intestinal microbial community is named gut microbiota (Shahi et al., 2017). The amount of microorganisms is 10^14^ with a total weight of approximately 2 kg (Picca et al., 2018). In human, distribution and species of the bacteria residing in the intestinal tract are always changing and uncertain, which depend on the physiological condition of the GI tract. So far, the exact species of microbe populations are unclear.

However, the comprehensive view of human-connected microbes has been offered by the Metagenomics of the Human Intestinal Tract and the Human Microbiome Project. There are a total of 2,172 species of microbes classified into 12 phyla in human, and *Proteobacteria, Firmicutes, Actinobacteria*, and *Bacteroidetes* phyla possess 93.5% of total microbes (Li et al., 2014; Hugon et al., 2015; Bilen et al., 2018). The phyla *Firmicutes* and *Bacteroidetes* are the majority, containing the genera *Prevotella*, *Bacteroides*, and *Ruminococcus*, as well as *Verrucomicrobia* and *Actinobacteria*, but *Proteobacteria* phyla members have a small quantity (Mowry and Glenn, 2018). Because many factors impact on gut microbiota, for example, genetic factors, sex, diet, and others, such as place of residence, smoking, etc., therefore, different ethnicities have different gut microbiomes (Blum, 2017).

### Role of the Gut Microbiota

The microbiota is involved in important homeostatic processes and essential for the homeostasis of intestinal intraepithelial lymphocytes (Liu L. et al., 2019). Besides the role of microbiota associated with GI function, the microbiota also contributes to inflammation and immune response, central and peripheral (enteric) neurotransmission, glucose metabolism, etc. (Liu et al., 2020). Therefore, the gut microbes play a beneficial role in maintaining homeostasis of immune systems of the host. The necessary vitamins and other substances involved in the development of the central nervous system (CNS) and immune regulation are produced by gut microbiota (Blum, 2017; Picca et al., 2018).

In a healthy organism, microbiota can also create a protective barrier against the infectious agents in the gut (Angelucci et al., 2019; Liu L. et al., 2019). Furthermore, a dynamic network is formed *via* the interaction among intestinal epithelial barrier, intestinal immune system, gut microbiota, and enteric nervous system (ENS) to coordinate the GI physiology and maintain homeostasis of gut (Pellegrini et al., 2018). The association of gut microbiota and its interaction with intestinal mucosal barrier and immune system in maintaining brain homeostasis have been demonstrated by more and more evidence (Foster et al., 2017; Fung et al., 2017).

### Impact of Dysbiosis of Gut Microbes on Body

In addition to destabilizing the intestinal environment, the dysbiosis of gut microbes can affect behavior, learning, and memory, as well as neurogenesis, etc. (Fang et al., 2016; Luczynski et al., 2016; Minato et al., 2017; Tremlett et al., 2017). Therefore, the gut microbiome plays a key role in maintaining the body healthy. So far, several review articles summarized well the role of gut microbiota in the maintenance of brain homeostasis (Fung et al., 2017; Tognini, 2017; Tremlett et al., 2017; Askarova et al., 2020). Here, we describe concisely the most important results about the role of gut microbiota in the regulation of brain physiological processes.

### Microbiota and Senescence

The relationship between the gut microbiota and the senescent brain is unclear, and until now, it is still an unanswerable question. As several neurodegenerative diseases occur in the elderly, it has attracted attention to the relationship between the gut microbiota and aging. However, at present, not many clinical and experimental studies evaluated this in the field. Claesson et al. (2012) studied the composition of the gut microbiota from 16 older than 65 years in the Ireland and showed more diverse gut microbiota with better health outcomes, indicating that the composition of the gut microbiota is closely related to health condition and immune function, and a diet rich in fruits and vegetables has a greater diversity of gut microbiota. Thus, one of the features of healthy aging may be the diversity of the gut microbiota (Claesson et al., 2012). Unfortunately, the study did not provide the information regarding the relationship between the gut microbiota and the senescence. The field is nascent, and so far, not many studies have been published.

The evidence concerning the relationship between gut microbiota and aging in mice showed that age-associated behavioral impairments were consistent with alterations of the microbiota (Scott et al., 2017), which is a direct evidence to confirm the close correlation between microbiota and aging. In the process of aging, the gut microbiota’s composition is altered, accompanied by increasing proteobacteria and decreasing probiotics, such as bifidobacteria, and neuroprotective molecules (Lambert et al., 2009; Caracciolo et al., 2014). The probiotic bacteria called “good” microbes play an advantageous role in human health and produce the essential substances to inhibit inflammation (Mukherjee et al., 2018).

The age-associated neuroinflammation, a crucial pathogenic factor in the development of AD and the cause or consequence of most neurodegenerative diseases, was ameliorated by administration with prebiotic inulin that targets the microbiota (Games et al., 1995). Microglial activation as an inflammatory hallmark in the pathology of AD is regulated by the microbiota, which plays a key role in aging and neurodegeneration (Abbas et al., 2002; Lambert et al., 2009). Moreover, high levels of proinflammatory cytokines in healthy elderly subjects were related to the disorder of microbiome function, particularly the genes encoding short-chain fatty acids (SCFAs; Claesson et al., 2012), which is a basic characteristic for the extensive age-related pathologies, such as age-related dysbiosis of gut microbes and neurological decline (Franceschi et al., 2000). Although lots of studies demonstrated this correlation, unfortunately a direct cause effect has not yet been established (Sun et al., 2019b; Kim et al., 2020). Thus, more studies are needed to show evidence of the relationship.

## Links Between Gut and Brain *via* Microbiome–Gut–Brain Axis

The gut microbiome is involved in bidirectional communication between the gut and brain, which is a significant scientific discovery recently (Erny et al., 2017; Fung, 2020). It has been suggested that human gut microbiome may be considered as the “second brain” and contributes to AD and other neurodegenerative disorders (Gershon, 1999; Schneider et al., 2019; Sochocka et al., 2019).

### Communication Between Brain and Gut

**Figure 1:**
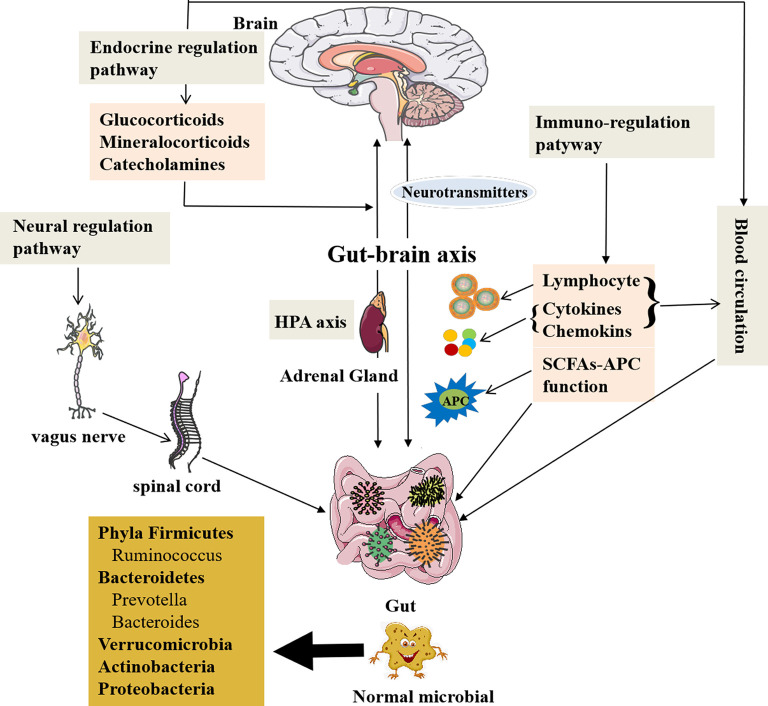
The role of the gut microbiota and communication with the brain. A healthy gut contains large fractions of the phyla Firmicutes and Bacteroidetes, including the genera *Prevotella*, *Bacteroides*, and *Ruminococcus* followed by *Verrucomicrobia* and *Actinobacteria*, but contains a low number of Proteobacteria phyla members (Mowry and Glenn, 2018). The gut microbiota is related to GI function, but also involved in several complex modulatory processes, inflammation and immune response, and peripheral (enteric) and central neurotransmission, as well as synthetize and secrete essential substances. The gut microbiota contributes to important homeostatic processes and is essential for the homeostasis of intestinal intraepithelial lymphocytes (Liu L. et al., 2019). The gut microbiota can affect brain function and bioactivity through gut–brain axis *via* several pathways: (1) the neural regulating pathway, in which vagus nerve links between the gut and the spinal cord (autonomic nervous system; Bonaz et al., 2018). The gut microbiota can secrete and regulate neurotransmitters of the CNS. (2) The endocrine pathway. The HPA axis can release glucocorticoids, etc., after stimulations by stress or other factors, which can alter gut microbiota composition and increase gut epithelium permeability and immune responses in gut (Ait-Belgnaoui et al., 2012; Park et al., 2013; Bellavance and Rivest, 2014). (3) The immune-regulating pathway *via* lymphocyte, cytokines, chemokines, and antigen-presenting effect of SCFAs communicating with the brain. (4) The blood circulation pathway (Logsdon et al., 2018). The immune and endocrine molecules, such as cytokines and hormones, can pass BBB and intestinal mucosa to influence both gut and brain functions (Zac-Varghese et al., 2010). BBB, blood–brain barrier; CNS, central nervous system; HPA axis, hypothalamic–pituitary–adrenal axis; SCFA, short-chain fatty acids; APC, antigen-presenting cells.

The gut microbiota can modulate brain signals and activity *via* the microbiome–gut–brain axis through the nervous, endocrine, and immune systems proven by many animal and preclinical experiments. Also the chemical substances produced by themselves (monoamines and amino acids) can cross the blood–brain barrier (BBB) reaching the CNS (Collins et al., 2012; Crane et al., 2015; Yano et al., 2015) and influence brain activity with possible repercussions on behavior (Wekerle, 2016; Kowalski and Mulak, 2019). The gut microbiota can also receive signals from the brain in the form of neurotransmitters, including acetylcholine, the modified amino acids glutamate and γ-aminobutyric acid (GABA), and the biogenic amines dopamine (DA), serotonin (5-HT), and histamine, interacting with the brain (Briguglio et al., 2018). Furthermore, the concept of the microbiome–gut–brain axis has been supported by the current research data; thus, the gut microbiome can communicate with the brain and is responsible for some neurodegenerative disorders (Haran et al., 2019). The new perspective makes us realize that the gut microbiota may play an important role in this mutual relationship between brain and gut communication, as well as physiological regulation. Figure 1 presents the microbiome–gut–brain axis containing several molecular pathways and their interactions. However, the microbiome–gut–brain axis is a complex multidirectional system between the gut microbiota, ENS, and the brain, which is still poorly understood.

#### Communication Through Neural Regulation Pathway

The pathways of communication between the gut and brain have been reported (Dinan and Cryan, 2017). The first pathway is neural regulation pathway, in which the vagus nerve links between the gut and the spinal cord (autonomic nervous system; Bonaz et al., 2018). The ended vagus nerve of brain stem nuclei receiving and giving afferent and efferent fibers may regulate the gut functions and send messages to other regions of CNS (Bonaz et al., 2018). The catecholamines or acetylcholine secreted from the brain affecting ENS circuits can modulate the gut functions (Mayer et al., 2015; Weinstein et al., 2015). It is also through the gut bacteria to exchange signals between ENS and CNS (Carabotti et al., 2015).

On the other hand, the gut microbiota is able to produce and modulate neurotransmitters in both CNS and peripheral nervous system, and intestinal environmental changes can affect lymphocytes of the gut to produce more cytokines and chemokines, such as interleukin 1 (IL-1), IL-6, IL-17, IL-22, tumor necrosis factor-α (TNF-α; Thaiss et al., 2016; Sochocka et al., 2019), and transforming growth factor β (Ma et al., 2017), as well as chemokine, fractalkine, and its receptor (CX3CR1; Merino et al., 2011), affecting the CNS through activating the endocrine or paracrine systems. The proinflammatory cytokines, IL-1, IL-6, IL-17, and TNF-α, are potentially harmful to the brain (Angelucci et al., 2019). The gut microbes secrete several important substances such as GABA, histamine, 5-HT, and DA, which contribute to neuroactive and immune regulation (Barrett et al., 2012; Lyte, 2013), and also produce toxic substances to the brain, such as ammonia and others (Galland, 2014). In addition, the microbiome–gut–brain axis can be affected by microbiota *via* immunological, neuroendocrine, and direct neural mechanisms (Logsdon et al., 2018), which insulted the brain to cause memory impairment, anxiety, and other cognitive dysfunctions (Gareau et al., 2011; Galland, 2014; Johnson and Foster, 2018) and resulted in several diseases, such as anxiety and depression (Lach et al., 2018; Capuco et al., 2020), neurodegenerative diseases, and drug-resistant epilepsy (Braakman and van Ingen, 2018).

#### Communication Through Endocrine Regulation Pathway

Endocrine regulating pathway is the second pathway of communication between the gut and brain. The hypothalamic–pituitary–adrenal (HPA) axis can release glucocorticoids, mineralocorticoids, or catecholamines after stimulations by stress or other factors, which result in the changes of intestinal microbiota components and the intestinal epithelium permeability, as well as immune responses in the gut (Ait-Belgnaoui et al., 2012; Park et al., 2013; Bellavance and Rivest, 2014). Enhanced genus *Clostridium* and declined *Bacteroides* as the feature of the gut dysbiosis were caused by high corticosterone levels in the stressed mice (Bailey et al., 2011). The glucocorticoids have both proinflammatory and anti-inflammatory roles; however, inflammations are related to damaged HPA axis functionality in AD and other neurodegenerative disorders (Silverman and Sternberg, 2012; Bellavance and Rivest, 2014; Hueston and Deak, 2014).

#### Communication Through Immune-Regulation Pathway

Immunoregulating pathway as the third pathway also participates in this communication between the gut and brain *via* gut microbes, which could affect antigen presentations and regulate cytokines production and lymphocyte function, as well as the development of two types of immune system through the gut–brain axis (Olszak et al., 2012; Fung et al., 2017). The gut microbiota also impacts on productions of SCFAs that can activate immune response and trigger inflammation in the brain, resulting in a series of neurological symptoms. Additionally, SCFAs are related to G-protein-coupled receptor 43 (GPR43) to lead a strong anti-inflammatory reaction (Maslowski et al., 2009). The gut microbes are necessary for host immunity generation in the GI tract. The data obtained from germ-free (GF) mice have shown that the maturations of the immune, endocrine, and nervous systems are affected by gut bacteria, which is a strong evidence for the gut microbiota linking with the brain *via* microbiome–gut–brain axis (Wang and Wang, 2016; Kowalski and Mulak, 2019).

#### Communication Through Blood Circulation

Communication between the gut and brain is also through the blood circulation (Logsdon et al., 2018). The cytokines and hormones as well as some molecules can pass BBB and intestinal mucosa to influence both gut and brain functions (Zac-Varghese et al., 2010). Furthermore, the central, peripheral, immune, and endocrine systems are involved in the communication between gut and brain in a multifunctional network formed by the microbiome–gut–brain axis (Borre et al., 2014).

However, the mechanisms that mediate gut–brain communication remain in its infancy. There are still many questions to explore, such as the molecular and cellular mechanisms underlying the microbiome–gut–brain axis in health and under pathological conditions, etc.

## Dysbiosis of Gut Microbes in AD Patients and Its Animal Models

Generally, the gut microbial communities in human are stable; however, they can be altered in the different conditions by the effects of various factors through their direct action (microbial infection) or indirect actions (antimicrobial protection hypothesis, hygiene hypothesis; DiSabato et al., 2016; Ashraf et al., 2019; Kong et al., 2020). Recently, the studies of several groups have been demonstrated that various diseases, including intestinal diseases and more systemic diseases such as diabetes, metabolic syndrome, and neurodegenerative disorders, including AD and others, are related to the imbalance of gut microbiota called “dysbiosis” (Del Tredici et al., 2002; Murono et al., 2015; Hu et al., 2016; Jiang et al., 2017). Occurrence and development of AD and other neurodegenerative disorders may be accompanied by the gut microbiome dysbiosis, inflammation, and dysfunction of the gut–brain axis. It has been speculated that AD may appear during the aging of immune system based on the theory of age-related dysbiosis derived from the association between gut microbiota and AD, which has been evidenced by clinical and experimental studies (Cattaneo et al., 2017; Pellegrini et al., 2018).

Generally, the traditional ecological measures are used to characterize the composition of the gut microbiome, including richness [the number of unique operational taxonomic units (OTUs) present in a participant], alpha diversity (the richness and abundance of OTUs within each participant), and beta diversity (the similarity or difference in composition between participants). Declined microbial richness and diversity as well as a distinct composition of the gut microbiome were found in AD patients. The levels of differentially abundant genera were correlated with cerebrospinal fluid (CSF) biomarkers of AD pathology (Vogt et al., 2017). In short, definite genera as more abundant in AD were related to greater AD pathology, whereas genera as less abundant in AD were associated with less AD pathology (Vogt et al., 2017).

As mentioned previously, immune response system participates in this communication between the gut microbes and brain. There is also a close interaction between gut microbes and the local as well as systemic immune system. In general, the gut dysbiosis could lead to dysfunctions of both innate and adoptive immune through several ways, such as changing antigen presentations, cytokines production, and lymphocyte functions, as well as increasing inflammation, etc., also can cause the gut–brain axis malfunction (Levy et al., 2017). In AD patients, the molecular and cellular alterations involving immune cells, such as T cells, B cells, microglia, etc., as well as immune mediators, occur not only in the peripheral blood, but also in the brain and the CSF, which may be associated with triggering immune response by the gut dysbiosis. The gut dysbiosis impacts on innate and adoptive immune response in AD patients obviously *via* activating immune/inflammatory cells, shifting them into inflammatory type to enhance immune mediated inflammatory response, and promoting neurodegeneration in the brain. The gut dysbiosis in AD was obviously correlated with more T helper 1 (T_H_1) cell infiltration into the brain (Togo et al., 2002; Monsonego et al., 2003), and increased T-cell infiltration in the brain parenchyma and peripheral T-cell responses to Aβ have been found in AD patients (Rogers et al., 1988; Monsonego et al., 2003).

Pathologically, deposition of Aβ plaques in the brain is a major character, and it has been considered as one of the important pathogenic factors in AD (Salter and Stevens, 2017; Angelucci et al., 2019). Reactive gliosis and neuroinflammation are the histological hallmarks and key factors in the pathogenesis of AD (Salter and Stevens, 2017; Yeh et al., 2017; Zhang et al., 2020). Microglial activation in the CNS is heterogeneous and categorized into two types: proinflammatory and anti-inflammatory microglia (Tang and Le, 2016; Yu et al., 2019). Microglia plays either a cytotoxic or neuroprotective role, depending on the types activated, which can be changed in the different stages of AD. The anti-inflammatory microglia phagocytizes Aβ plaques by the Fc receptors and promotes the cleaning and degradation of Aβ by possibly increased phagocytic and lysosomal activity, as well as restriction of the inflammatory response (Kamphuis et al., 2016; Dubbelaar et al., 2018). Oppositely, proinflammatory microglia leads to Aβ accumulation, inducing cell death and worsening disease (Fakhoury, 2018). We speculated that microglial malfunction may be the basis of AD pathogenesis and precede and accelerate the onset of AD.

Table 1 compares the gut microbiota between healthy subjects and AD patients, in which alterations of the gut microbiota can be seen in the AD patients; thus, dysbiosis of gut microbes may be involved in AD-related impairments.

**Table 1:** The comparisons of the gut microbiota between healthy subjects and Alzheimer’s disease (AD) patients.

	Healthy	AD	References	subjects	patients	
The gut microbiota	Eubacterium rectale	**↑**	Cattaneo et al. (2017)
	Escherichia/Shigella	**↓**	
	Bacteroides	**↓**	Zhuang et al. (2018)
	Actinobacteria	**↑**	
	Bacilli	**↑**	
	Negativicutes	**↓**	
	Ruminococcaceae	**↑**	
	Enterococcaceae	**↑**	
	Lactobacillaceae	**↑**	
	Lanchnospiraceae	**↓**	
	Veillonellaceae	**↓**	
	Firmicutes	**↓**	Vogt et al. (2017)
	Bifidobacterium	**↓**	
	Dialister	**↓**	
	Bacteroidetes	**↑**	
	Blautia	**↑**	
	Phylum Firmicutes	**↓**	Liu et al. (2019)
	Proteobacteria	**↑**	
	Gammaproteobacteria	**↑**	
	Enterobacteriales	**↑**	
	Enterobacteriaceae	**↑**	

*↑:increase; ↓:decrease*.

### Dysbiosis of Gut Microbes Involved in the Pathogenesis of AD Models

So far, the evidence obtained about the role of dysbiosis of gut microbes in AD pathophysiology is mainly from its animal models. A significant decrease in the Aβ pathology was observed in GF mice, and after the control mice were exposed to the gut microbiota, the Aβ pathology occurred again (Harach et al., 2017). In addition, an obvious absence of amyloid plaque deposit and neuroinflammation were seen in GF mice when microbes were not present (Harach et al., 2017). Of course, the pathological manifestations in GF mice may not be completely attributed to gut dysbiosis, because GF mice also exhibited defects in the immune system and difficulties with energy acquisition, etc., which also impact on the pathological changes in GF mice. The changes of gut microbiota promoted Aβ protein accumulations in the gut. Evidently, a thoroughly changed gut microbiome was found in APP transgenic (Tg) mice (AβAPP) [a genetic model of AD; the mice overexpress mutated forms of human amyloid precursor protein (APP) linked to familial AD] when compared to wild-type mice (WT; Wang X.-L. et al., 2015). The removal of gut microbiome was related to central Aβ levels in AD mice; however, increased amyloid accumulation was found in the brain after transplantation by microbiota from AD mice (Harach et al., 2017). Similarly, high levels of Aβ protein of brain and related behavioral alterations were associated with the gut dysbiosis in APP/PS1 mice [a genetic model of AD; APP/PS1 mice are double Tg mice expressing a chimeric mouse/human APP (Mo/HuAPP695swe) and a mutant human presenilin 1 (PS1-dE9); Shen et al., 2017]. Moreover, both enhanced Aβ protein precursor (AβPP) accumulation in the gut and Firmicutes/Bacteroidetes ratio were found in 5xFAD mice (a transgenetic model of AD; 5xFAD mice express human APP and PSEN1 transgenes with a total of five AD-linked mutations: APP KM670/671NL, APP I716V, APP V7171, and PSEN1M146L and PSEN1L286V) following the change of the gut microbiota composition in these mice since the earliest phase of the diseases (Brandscheid et al., 2017). These data suggest that changing of gut microbiome in the animal models of AD promotes deposit of Aβ protein in the brain.

A significant study indicated that calorie restriction decreased Aβ deposition in the brain of AD mouse model. During aging process, calorie restriction could change the gut microbiome, including an increase in *Bacteroides*, which was found obviously in female Tg2576 mouse model when compared to WT mice. It has been demonstrated that the specific gut microbiota change was related to Aβ levels, and the change had a greater impact on females than males. Furthermore, long-term calorie restriction can change the gut environment and prevent the expansion of microbes that promotes age-related cognitive decline (Cox et al., 2019).

Interestingly, there was activation of immune/inflammatory cells and high expressions of Aβ and phosphorylated tau (p-tau) protein, as well as neuronal coding rearrangements in the gut of APP/PS1 mice, which feature is accompanied by lower levels of neuronal nitric oxide synthase and choline acetyltransferase, suggesting that Aβ and p-tau protein deposits in the gut can influence local and peripheral neurogenic/inflammatory responses and promote inflammation and neurodegeneration in the brain of AD models (Haghikia et al., 2016; Feng et al., 2018). It has been shown that enhanced Aβ protein expression in the gut precedes inflammation in the brain of TgCRND8 mice (a genetic model of AD; TgCRND8 mice overexpress mutant human APP KM670/671NL and APP V717F; Semar et al., 2013). Aβ protein can also be transmitted to the CNS through myenteric neurons and nerve gut–brain axis involved in the pathogenesis of AD directly (Zhao and Lukiw, 2015; Pistollato et al., 2016). However, the causal relationship between these possible pathogenic factors is unclear; therefore, further studies are needed to investigate.

### Dysbiosis of Gut Microbes Associated With Inflammation in AD Models

It is beyond doubt and has been evidenced that inflammation is a crucial factor in the pathogenesis of AD. Recent studies have shown that a strong correlation between NLRP3 inflammasomes, one of the multiprotein complexes, and initiation of inflammation and neurological diseases, is identified (Pellegrini et al., 2019). NLRP3 inflammasomes are key molecules in neuroinflammation and Aβ caused AD pathology in AD models (Heneka et al., 2013; Ising et al., 2019). Conversely, impaired NLRP3 inflammasome function lowered tau hyperphosphorylation by regulating tau kinases and phosphatases (Ising et al., 2019; Tejera et al., 2019). NLRP3 knockout (KO) mice exhibited significant difference of the composition of gut microbiota and behaviors compared with WT mice, suggesting that NLRP3 inflammasome deficiency affected the gut microbiota composition (Zhang et al., 2019). Transplantation of the gut microbiota of NLRP3 KO mice or using NLRP3’s inhibitor ameliorated depressive-like behaviors *via* remodeling gut microbiota (Zhang et al., 2019). The cognitive function of AD mice was repaired by using NLRP3 inhibitor, which may be associated with altering gut microbiota (Daniels et al., 2016; Ising et al., 2019; Tejera et al., 2019). Inflammation playing a central role in AD is linked to the closed relationship between gut microbiota and AD (Calsolaro and Edison, 2016).

### Dysbiosis of Gut Microbes and Enteric Inflammation or Infections in AD Patients

In AD patients, the proportion and prevalence of bacteria synthesizing butyrate were low, and the abundances of taxa were high that lead to inflammation compared to healthy or other dementia types, which evidenced that the nexus between the gut microbiome and an altered epithelial homeostasis could have an effect on AD (Daniels et al., 2016) by increases in inflammatory and decreases in anti-inflammatory microbial metabolism (Haran et al., 2019).

Several studies on dysbiosis of gut microbes in AD patients have exhibited that AD’s main pathological features in the brain, such as amyloidosis and inflammation, are linked to inflammatory bacteria and their neurotoxic products, like lipopolysaccharides (Bester et al., 2015; Cattaneo et al., 2017). In AD patients, increased *Bacteroides* and *Blautia* and decreased relative abundance of the genera *SMB53* and *Dialister* were a feature of the changes of gut microbiota, which was associated with high levels of chitinase-3-like protein 1 and p-tau, accompanied by a low Aβ42/Aβ40 ratio in CSF (Vogt et al., 2017). Intestinal inflammation in AD patients was positively correlated with a high level of fecal calprotectin (Leblhuber et al., 2015). However, the clinical evidence on accumulations of Aβ protein, AβPP, and p-tau in the gut of AD patients is rare, and the data obtained are contradictory (Joachim et al., 1989; Puig et al., 2015). Meanwhile, studies with no matched healthy controls are also unable to make such conclusion; i.e., there is a causal relationship between intestinal Aβ and p-tau deposition, inflammation and gut dysbiosis.

The chronic *Helicobacter* *pylori* infection can trigger the release of inflammatory mediators and is associated with low Mini-Mental State Examination score in AD patients when compared with patients without infections (Roubaud-Baudron et al., 2012).

Infections by *H. pylori*, *Borrelia burgdorferi*, and *Chlamydia pneumoniae*, and so on, increased levels of Aβ40 and Aβ42 in serum of AD patients (Bu et al., 2015). *In vitro*, the neuroblastoma cell cultures treated by *H. pylori* filtrate induced tau hyperphosphorylation, which was similar to AD tau pathological changes (Wang X.-L. et al., 2015). Furthermore, the inflammatory disorders are also linked to gut dysbiosis caused by viruses, such as herpes simplex virus type 1, which can be one of the crucial risk factors for AD. Maintaining the homeostasis of intestinal intraepithelial lymphocytes required these commensal viruses; however, the sustaining intestinal homeostasis can be also destroyed by infections with bacteria and virus (Harris and Harris, 2015). Collectively, the gut dysbiosis could be a risk factor for AD due to lacking or reducing immune defenses in the seniors (Angelucci et al., 2019).

Although gut dysbiosis contributes to the pathogenesis of many neurological and neurodegenerative diseases generally, the types of microbiota changes in the gut of AD are different from those of other neurodegenerative diseases, when compared with multiple sclerosis (MS), Parkinson disease (PD), and amyotrophic lateral sclerosis as shown in Table 2. We speculated that some microbial changes are relatively specific to AD due to causing AD pathology different from other diseases. Increased *Bacteroides* was found in the AD patients, and *Bacteroides* colonization aggravated Aβ deposition, which is speculated to be a mechanism whereby the gut impacts AD pathogenesis (Cox et al., 2019). However, there are too many factors affecting the gut microbiota; it is difficult to determine a causal relationship and needs to be further explored.

**Table 2:** The gut microbes in central nervous system (CNS) disorders and treatments by microbes or microbial products.

CNS disorders	Pathologies	Gut microbes ↑	Gut microbes ↓	Treatments	Other results
AD	Glial cell activation and inflammatory molecules production. Aβ plaques containing extracellularly deposited Aβ. Intracellular neurofibrillary tangles formed with hyperphosphorylated and misfolded tau protein (Angelucci et al., 2019).	*CMV, HSV-1, B. burgdorferi, C. pneumoniae and H. pylori* (Kountouras et al., 2009; Bu et al., 2015) *Bacteroides*, *Gemellaceae, genera Blautia, Phascolarctobacterium, and Gemella* (Vogt et al., 2017).	*Firmicutes, Actinobacteria, genera* SMB53 (family *Clostridiaceae*), and *Dialister, Clostridium, Turicibacter*, and cc115 (family *Erysipelotrichaceae*; Vogt et al., 2017).	Increased MMSE after treatments by probiotic (*Lactobacillus acidophilus, Lactobacillus casei, Bifidobacterium bifidum, and Lactobacillus fermentum*, Akbari et al., 2016), or by selenium plus probiotic (Tamtaji et al., 2019a). GV-971 had cognition improvement by targeting gut dysbiosis (Wang et al., 2019). Increased MMSE after triple eradication regimen (omeprazole, clarithromycin and amoxicillin) treatment for HP+ patients (Kountouras et al., 2009).
ALS	Tetanus and botulinum toxins, “leaky gut”; higher richness of OTUs and reduction of butyrate (Zhu et al., 2020).	*Dorea* (Fang et al., 2016). *E. coli and enterobacteria* (Mazzini et al., 2018).	*Oscillibacter, Anaerostipes, Lachnospiraceae* (Fang et al., 2016). Total yeast (Mazzini et al., 2018).
MS	BBB integrity disruption and astrocyte pathogenicity, primary demyelination, axonal loss, and reactive gliosis in the CNS (Duffy et al., 2014; Chu et al., 2018).	*Methanobrevibacter* and *Akkermansia* (Jangi et al., 2016). *Actinobacteria,Bifidobacterium* and *Streptococcus* (Miyake et al., 2015) *Firmicutes, Archaea Euryarchaeota* (Tremlett et al., 2016) *Ruminococcus* (Cantarel et al., 2015)	*Butyricimonas* (Jangi et al., 2016) *Bacteroides, Faecalibacterium, Prevotella, Anaerostipes Clostridia* XIVa and IV *Clusters* (Miyake et al., 2015) *Fusobacteria* (Tremlett et al., 2016) *Faecalibacterium, Bacteroidaceae* (Cantarel et al., 2015)	*Prevotella, Sutterella*↑ and *Sarcina*↓ after IFN-ß or GA treatmet (Jangi et al., 2016). *Faecalibacterium*↑ after GA treatment (Cantarel et al., 2015). *C. perfringens* ↓ after Fingolimod, DMF or Teriflunomide treatment (Rumah et al., 2017).	Absence (vs. presence) of *Fusobacteria* associated with relapse risk (Tremlett et al., 2016). Inhibiting the growth of *C*. *perfringens* enhancing the efficacy of MS drugs (Rumah et al., 2017).
PD	Higher frequency of α-synuclein, dopaminergic neuronal loss (Zhu et al., 2020).	*Prevotellaceae* (Scheperjans et al., 2015). *Blautia, Coprococcus, and Roseburia* (Zhu et al., 2020).	*H. pylori* (Fasano et al., 2015). *E. coli, Ralstonia, Oscillospira and Bacteroides* (Zhu et al., 2020).	Decreased MDS-UPDRS after probiotic (*Lactobacillus acidophilus, Bifidobacterium bifidum, Lactobacillus reuteri, and Lactobacillus fermentum*) treatment for 12 weeks (Tamtaji et al., 2019b).	Reduced *Prevotellaceae* significantly in PD as a sensitive biomarker for PD (Zhu et al., 2020).

*Abbreviations: Aβ, Amyloid β; AD, Alzheimer’s disease; ALS, Amyotrophic lateral sclerosis; BBB, Blood-brain barrier; CNS, Central nervous system; DMF, Dimethyl fumarate; GA, Glatiramer acetate; GV-971, Sodium oligomannate; HP+, Helicobacter pylori positive; MDS-UPDRS, Movement disorder society unified Parkinson’s disease rating scale; MS, Multiple sclerosis; MMSE, Mini-mental state examination; OTUs, Operational taxonomic units; PD, Parkinson’s disease*. ↑:increase; ↓:decrease.

Up to now, no study confirmed clearly that Aβ deposition or tau accumulation is related to alter a particular microbe in the gut of AD patients. However, the specific microbes resulting in inflammation may promote Aβ and p-tau protein deposits in the gut indirectly, and the two promote each other. We considered that the infections by *H. pylori* and *B. burgdorferi* and enhanced *Bacteroides* may promote Aβ deposition or tau accumulation in the gut of AD patients, which needed to be evidenced in the future studies.

### Factors Caused Dysbiosis of Gut Microbes Can Target AD

The factors that disturbed the gut microbiota to lead gut dysbiosis could target AD (Stenman et al., 2013; Vogt et al., 2018; MahmoudianDehkordi et al., 2019). There was an interaction between gut microbiome and bile acid (BA) levels. The bacteria containing the abundant bile salt hydrolase can easily change BA pattern to modulate the commensal bacteria and protect the integrity of the intestinal barrier (Stenman et al., 2013; Shapiro et al., 2014; MahmoudianDehkordi et al., 2019). In the CSF of mild cognitive impairment (MCI) and AD patients, the high level of trimethylamine *n*-oxide (TMAO), a metabolite derived from gut microbiota, was related to the biomarkers of AD in CSF (Vogt et al., 2018).

A pivotal pathogenic factor, oxidative stress (OS) has been shown to contribute to the development of AD. In the CNS, the reactive oxygen species (ROS) levels and inflammation can be enhanced by a microbiota type to favors abnormal aggregation of Aβ, which speculated that high levels of CNS OS may be due to gut dysbiosis or its consequence (Jones et al., 2012; Dumitrescu et al., 2018). In short, it can be seen from the above results and summarized as follows: (1) chronic bacterial infections as a possible etiology linking AD pathogenesis; (2) obvious alteration in the compositions of gut microbiome in AD; and (3) rising proinflammatory and lowering anti-inflammatory bacteria in the gut related to systemic inflammation in the patients suffering from the brain amyloidosis and cognitive impairment, which changes might impact brain functions. Thus, microbial dysbiosis or imbalance may potentially contribute to the pathogenesis of AD.

## Is Targeting Dysbiosis of Gut Microbes as a Therapeutic Manipulation in AD?

The gut microbiota may impact on AD development and progress as descripted above. Dysbiosis of gut microbes supposes to be involved in the pathogenesis of AD. Despite much disappointment in anti-AD drug discovery previously, it is still promising and possible to find new treatments basis on gut microbe impacting on AD. Modifying the microbiota composition or remodeling gut microbes using the substances or manipulations that are able to change their composition or balance gut microbes, such as antibiotics and others, may affect or provide therapy for AD and other neurological diseases (Chu et al., 2018).

### Targeting Dysbiosis of Gut Microbes by Antibiotics in AD Patients and Its Models

Usually, eliminating and avoiding bacterial colonization are the main effects of antibiotics on human, rather than targeting the specific types of bacteria (Angelucci et al., 2019). After treatments by the broad-spectrum antibiotics, the composition of the gut microbiota was markedly altered, and its biodiversity was declined, as well as the colonization was temporized (Angelucci et al., 2019). Therapies with antibiotic could change the gut microbiota during the different length of time (Ianiro et al., 2016) and alter behavior we know well as brain chemistry in both humans and animals (Jernberg et al., 2007; Fröhlich et al., 2016). However, the evidences from two studies displayed that the antibiotic treatment caused also neuropsychiatric symptoms such as anxiety, psychosis, and delirium in AD patients who received antibiotic as a cocktail therapy (Loeb et al., 2004; Molloy et al., 2013), which is associated with antibiotic treatment of *H. pylori* infections, but these neuropsychiatric symptoms as side effects were not found in the general population (Neufeld et al., 2017). The effects of antibiotics on AD may be extensive or even opposite, depending on the antibiotics applied and on the role of targeted gut microbiome in the pathogenesis of AD. The antibiotic therapy was effective in the animal models of AD, but it has not yet been widely investigated in AD patients, because it is not clear which microbiomes dominate in the gut of AD patients and whether there are safe antibiotics available (Panza et al., 2019). Besides, there is a lack of such study on the effect of different antibiotics on AD pathology; further study is needed.

Obviously, lower amounts of microglia and astrocyte accumulation around amyloid plaques in the hippocampus and reduced insoluble Aβ plaques in aged APPSWE/PS1DeltaE9 Tg mice of AD model (which overexpress the Swedish mutation of APP KM670/671NL together with PS1 deleted in exon 9) after treatment with an antibiotic cocktail (Minter et al., 2017) were found, which is only circumstantial evidence that antibiotic interfered with microglial activation through reducing the amounts of microglia. However, in APP/PS1 Tg mice, the treatments with cocktail of antibiotics resulted in enhanced neuroinflammation and proinflammatory cytokine levels, and the disease itself was deteriorated (Minter et al., 2016). The harmful effects of antibiotics may break down the balance of gut bacteria, as streptozotocin and ampicillin did, which favors AD or worsens its course (Zarrinpar et al., 2018). Ampicillin increased rat serum corticosterone related to memory dysfunctions and decreased brain-derived neurotrophic factor in hippocampus, the features of AD pathology. Also, ampicillin deteriorated the anxiety-like behavior and impaired spatial memory in rats (Fröhlich et al., 2016). Surprisingly, the disorder of physiological and psychological function caused by ampicillin in rats was turned down by administration with probiotics (Wang T. et al., 2015). The clinical and experimental studies highlight that the results using antibiotics targeting and remodeling gut microbes in AD patients are controversial. Also there were some adverse consequences after antibiotic treatment, such as gut microbes coming back with their same features. According to the results of the current studies, it might be difficult to determine the effect of antibiotics in the treatment of AD. Attention should be paid to protection of the new beneficial and specific microbes and to the focus of future therapeutic trials by antibiotics in AD.

### Remodeling Gut Microbes by Fecal Microbiota Transplantation

A new therapeutic method with fecal microbiota transplantation (FMT) has been applied in the neurodegenerative disorders and their animal models, as well as other diseases recently (Allegretti et al., 2018). FMT consists of obtaining a fecal specimen from a healthy donor and administering a sample through either the mouth or the rectum of the ill person. The obtained results from FMT were encouraging and remarkably good in patients with recurrent *Clostridium* *difficile* infection, and FMT has become an important care option. FMT improved clinical symptoms obviously and fecal microbiome in the dog model with inflammatory bowel disease (Niina et al., 2019). In this respect, most clinical and experimental studies have been done in the patients with PD and its animal model. The exciting results with slowing down clinical progress in PD patients were obtained after reconstruction of the gut microbiome by FMT (Dutta et al., 2019). A study reported that constipation in a PD patient was clearly relieved after FMT through reconstruction of gut microbiota (Huang et al., 2019). The mechanisms behind the therapeutic effects of FMT are related to significant reduction of gut microbial dysbiosis and fecal SCFAs, as well as increment of levels of striatal DA and 5-HT, which has been evidenced in PD mice model. Furthermore, the activation of glial cells in the substantia nigra and TLR4/TNF-α signaling pathway molecules was inhibited by FMT in both gut and brain, which further evidences that gut microbial dysbiosis contributes to PD development, and FMT is beneficial to PD models (Sun et al., 2018). Therefore, the gut microbiota reconstruction may have therapeutic effects on PD patients and is a new therapeutic option (Fang, 2019). However, the study on treatment with FMT in AD and its animal models is scarce.

Recently, DeFilipp et al. (2019) reported a patient death treated with FMT due to extended-spectrum beta-lactamase-producing *Escherichia coli* bacteremia. To avoid similar accidents, it is necessary to enhance donor screening in order to reduce the transmission of microorganisms when treating patients with FMT and to properly evaluate the benefits and risks of FMT in different patient populations (DeFilipp et al., 2019), which should improve the new approaches for treatments in AD patients in the future (Blaser, 2019).

### Remodeling Gut Microbes With Substances/Compounds in AD Patients and Its Models

Moreover, prebiotic fructooligosaccharides (FOSs) as dietary supplements ameliorated cognitive deficits and pathological changes in the APPSWE/PS1DeltaE9 Tg mice and increased the levels of synapsin I and synaptic plasticity markers, postsynaptic density protein 95, and decreased the phosphorylated level of c-Jun N-terminal kinase, indicating that FOS can modulate the gut microbiota-glucagon-like peptide-1 (GLP-1)/GLP-1 receptor (GLP-1R) pathway to play a beneficial role in AD (Sun et al., 2019a).

However, the results obtained from a double blind clinical trial, which was carried out in AD patients treated by probiotic supplementation (PS) and placebo, respectively, for 12 weeks, were negative, indicating that treatment with PS was ineffective in the severe AD patients, and the curative effect with PS was related to severity of AD at least (Agahi et al., 2018). In another clinical trial, treatment with multispecies probiotic for 4 weeks changed gut microbiota composition and tryptophan metabolism in serum of AD patients. Furthermore, a correlation between kynurenine/tryptophan and neopterin levels was observed indicating activation of macrophages and/or dendritic cells in AD patients (Leblhuber et al., 2018).

An exciting new drug for treatment of AD named GV-971 (sodium oligomannate) is discovered more recently (Wang et al., 2019). Polysaccharides or oligosaccharides are able to regulate gut microbiota (Thomson et al., 2018). Main therapeutic effects of GV-971 on AD are: (1) restoring the balance of gut microbiota *via* targeting and remodeling gut microbiota; and (2) inhibiting the neuroinflammation caused by gut bacterial amino acids to slow down AD progression (Wang et al., 2019). The enhanced levels of phenylalanine and isoleucine in periphery caused by dysbiosis of the gut microbiota can induce activation of proinflammatory microglia and proliferation of infiltrated inflammatory T_H_1 cells from blood into the brain of AD mice deteriorating inflammation. Meanwhile, there were high levels of phenylalanine, isoleucine, and T_H_1 cell in blood of MCI patients. The oligomannate sodium GV-971 is a carbohydrate-based anti-AD drug that markedly improved cognition in Chinese patients by targeting gut dysbiosis, dropping phenylalanine/isoleucine in the feces and blood, and inhibiting T_H_1-associated neuroinflammation in the brain to reverse the cognition impairment (Wang et al., 2019).

**Figure 2:**
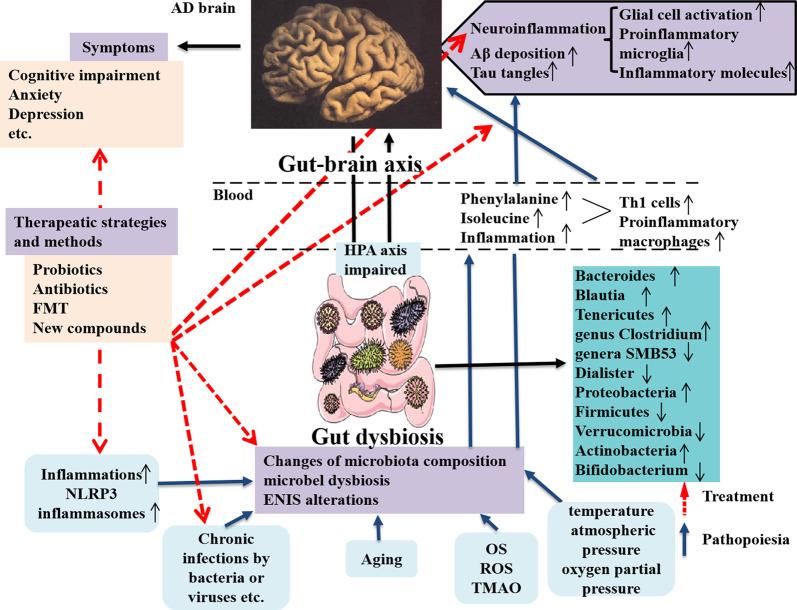
Dysbiosis of gut microbes in AD and the intervention strategy. The communications between the gut microbes and brain is through gut–brain axis to involve in the modulatory processes, inflammation, and immune response to maintain homeostasis of body. The gut microbiota can affect brain function and bioactivity (Liu L. et al., 2019). Several factors including inflammation (such as NLPR3 inflammasomes), chronic infections by bacteria or viruses, aging, and increased inflammatory molecules (OS, ROS, and TMAO) production, etc., can cause the changes of microbiota composition, microbial dysbiosis, and ENIS alterations, which contribute to AD pathology in the brain, including neuroinflammation due to glial cell and M1 microglia activations and increased inflammatory molecules production and enhanced Aβ deposition and tau tangles in the brain, these being the classical pathological features in AD (Angelucci et al., 2019). The microbial dysbiosis also leads to the peripheral accumulation of phenylalanine and isoleucine, which promotes the differentiation and proliferation of proinflammatory T helper 1 cells that infiltrated into the brain of AD mice *via* blood circulation, associated with the M1 microglia activation, contributing to neuroinflammation in AD (Liu P. et al., 2019). AD pathological changes result in a series of clinical symptoms, as cognitive impairment, anxiety, depression, and others. The novel intervention strategies contain applying probiotics, antibiotics, and FMT, as well as using a compound, such as GV-971 and others, which are targeting and remodeling gut microbiota and suppressing gut bacterial amino acids-shaping neuroinflammation to inhibit AD progression. AD, Alzheimer disease; OS, oxidative stress; ROS, reactive oxygen species; TMAO, trimethylamine *n*-oxide; ENIS, enteric neuroimmune system; FMT, fecal microbiota transplantation.

In the study, the authors focused on the gut microbiota associated with neuroinflammation in AD patients and animal models through observing T_H_1 and proinflammatory microglia activities. They showed that alterations of gut microbiota composition in AD were obviously correlated with more T_H_1 cell infiltration into the brain. Also removing the gut microbiota by administrating antibiotic in AD mice can block T_H_1 cell infiltration and proinflammatory microglia activation. Additionally, strengthened T_H_1 cell infiltration and proinflammatory microglia activation in WT mice can be caused by FMT from AD mice and prolonged contact with fecal bacteria (Wang et al., 2019). By contrary, less T_H_1 cell infiltration can be seen in Tg mice receiving FMT from WT mice (Wang et al., 2019). The new discoveries emphasize the abnormal phenylalanine and isoleucine induced by gut microbiota worsening T_H_1 cell–mediated inflammation in AD and its models and effectively remodeling the gut microbiota is a novel strategy for AD therapy (Figure 2). The therapeutic strategies targeting the gut microbiota in AD patients and animal models are summarized in Table 3. Unfortunately, it is still difficult to determine which microbes are special targets for AD therapy currently, because many factors, including diet, place of residence, smoking, ethnicity, etc., can also influence the changes of the gut microbiota in AD.

**Table 3:** Therapeutic strategies targeting the gut microbiota in AD animal models and patients.

Pre-clinical and clinical studies	Therapeutic manipulations	Gut microbiota changes after treatments	Results	References
APP_SWE_/PS1_deltaE9_	ABX	*Lachnospiraceae and S24-7↑*	Brain Aβ deposition↓ Microglia and astrocyte accumulation around amyloid plaques↓ Blood: Foxp3+ T-regulatory cells ↑	Minter et al. (2017)
APP_SWE_/PS1_deltaE9_	ABX	*Akkermansia* ↑ *Lachnospiraceae↑*	Brain Aβ deposition↓ Brain soluble Aβ↑ Blood: CCL11, CXCL16, LIX, TIMP-1 and NFαR1↑	Minter et al. (2016)
APP/PS1 mice	probiotic	*Eubacteria, Roseburia*↑ *Clostridium↓*	Spatial memory↑ Hippocampus Aβ plaques↓ The numbers of microglia in hippocampus ↓ OOG1 in the hippocampus↓	Abraham et al. (2019)
APP/PS1 mice	OMO	*Lactobacillus, Akkermansia, Bacteroides, Adlercreutzia*, and *Desulfovibrio*↑ *Ruminococcus, Bifidobacterium, Blautia, Oscillospira, Coprococcus, Sutterella*, and *Clostridium*↓	Learning and memory impairments↓ Aβ_1–42_ positive cells in brain↓	Xin et al. (2018)
APP/PS1 mice	FOS	*Epsionproteobacteria, Proteobacteria, Helicobacteraceae, Deferribacteraceae, Helicobacter*↓ *Actinobacteria, Lactobacillus↑*	Cognitive deficits↓ Aβ deposition in the brain↓ synapsin I and PSD-95 in the brain ↑ phosphorylated level of JNK in the brain↓ GLP-1, GLP-1R in the gut↑	Sun et al. (2019a)
5XFAD mice	GV-971	*remodeling the gut microbiota*	Cognitive impairment↓ Aβ and tau phosphorylation in the hippocampus ↓ Brain Th1 cells↓ IBA1 in hippocampal↓ Inhibiting neuroinflammation by harnessing amino acid metabolism	Wang et al. (2019)
D-galactose and Aβ_1__-__42_-induced Alzheimer’s rats	OMO	*Firmicutes, Bacteroidetes, Bacteroidia, Bacilli, Lactobacillales, Bacteroidales, Lactobacillaceaes*↑ *Clostridia, Clostridiales↓*	Learning and memory abilities↑ Swelling of brain tissues and neuronal apoptosis↓ Tau and Aβ_1__-__42_ expression in brain↓ Tissue damages and inflammation induced by TNBS ↓	Chen et al. (2017)
ddY mice (the strain is outbred and has been maintained as a closed colony and a model of postprandial hypertriglyceridemia in response to dietary fat)	B. breve A1	*Phylum Actinobacteria and Bifidobacteriaceae*↑ *Odoribacteraceae and Lachnospiraceae↓*	Prevents Aβ-induced cognitive dysfunction Aβ-induced gene expression changes in the hippocampus↓ Plasma acetate↑	Kobayashi et al. (2017)
D-Galactose-induced Alzheimer’s rats.	*Lactobacillus plantarum* MTCC1325		Cognition deficits↓ Aβ in brain↓ Ach and AChE in hippocampus and cerebral cortex↑	Nimgampalle and Kuna (2017)
Wistar rats intrahippocampal injection of Aβ_1–42_	*Lactobacillus acidophilus*, *L. fermentum*, *Bifidobacterium lactis*, and *B. longum*	*Coliform*↓ *Bifidobacterial* and *lactobacilli*↑	Learning and memory abilities↑ MDA in the hippocampus ↓ SOD in the hippocampus ↓	Athari Nik Azm et al. (2018)
AD patients	Probiotic (*Lactobacillus acidophilus*, *Lactobacillus casei*, *Bifidobacterium bifidum*, and *Lactobacillus fermentum)*		MMSE score↑ hs-CRP, HOMA-IR, HOMA-B and MDA in blood↓ QUICKI↑	Akbari et al. (2016)
AD patients	selenium plus probiotic *(Lactobacillus acidophilus, Bifidobacterium bifidum*, and *Bifidobacterium longum)*		MMSE score↑ hs-CRP, HOMA-IR, LDL-cholesterol, total-/HDL-cholesterol ratio, and QUICKI in blood↓	Tamtaji et al. (2019a)

*Abbreviations: ABX, broad-spectrum antibiotics, including gentamicin, vancomycin, metronidazole, neomycin, ampicillin, kanamycin, colistinand and cefaperazone; Ache, Acetylcholinesterase; B. breve A1, *Bifidobacterium breve* strain A1; FOS, Prebiotic fructooligosaccharides; GLP-1, Glucagon-like peptide-1; GLP-1R, GLP-1 receptor; GV-971, Sodium oligomannate; HOMA-B, Homeostatic model assessment for B-cell function; HOMA-IR, Homeostatic model of assessment for insulin resistance; hs-CRP, High sensitivity C-reactive protein; JNK, C-Jun N-terminal kinase; MDA, Malondialdehyde; OMO, Oligosaccharides from Morinda officinalis; OOG1, 8-oxoguanine DNA glycosylase-1; PSD-95, Postsynaptic density protein 95; QUICKI, Quantitative insulin sensitivity check index; SOD, Superoxide dismutase*. ↑:increase; ↓:decrease.

Targeting dysbiosis of gut microbes as a future therapeutic manipulation in many diseases including AD is a promising therapeutic strategy. The advantages of this manipulation are effective to treat or alleviate diseases. It prevented the recurrence of MS by adding microbes to the daily diet for long-term use (Tremlett et al., 2016) and slowing down the clinical progress in PD patients (Dutta et al., 2019), as well as treated refractory constipation in a PD patient (Huang et al., 2019), etc., in which the conventional treatments were helpless. This therapeutic manipulation overcomes the disadvantages of conventional treatments that either overlook the microbes in the mechanism of action or remove vast populations of microbes *via* antibiotics. It aims at rebalancing the gut microbiota balance, preventing dysbiosis, and keeping the internal environment stable. Thus, the therapeutic manipulation is exciting and challenging. However, there are still many problems and disadvantages in microbial treatment. First, the obtained results treated by microbes or microbial products in human disorders are contradictory. Also the sample size treated by microbes is too small, which causes doubt to the effectiveness of the treatments. Second, it is difficult to select the correct therapeutic scheme. Third, although the side effects of the microbial treatment were reported sporadically, the exact side effects were not found because of the small sample size. It is unclear whether microbial therapy can cause other diseases.

There are many ways to balance the microbiota in the gut, such as reducing or inhibiting infections and inflammation of the system and gut, eating a healthy diet, quitting smoking, increasing immunity, and exercise, etc.; thus, balancing the microbiota can be maintained for a long time.

## Conclusions

The gut microbiome may contribute to the pathogenesis of AD and neurodegenerative disorders through the microbiota–gut–brain axis pathway. The dysbiosis of the gut microbiome and resulting inflammation may be important pathogenic factors in development and progression of AD. Targeting and remodeling the gut microbiome open a potential new door to an effective therapeutic strategy in AD patients. However, the field is nascent, and the data obtained are controversial, as well as many factors influence the gut microbiome. It is still difficult to establish the relationship between the gut microbiome and brain bioactivity in any specific disorder of humans *via* the microbiota–gut–brain axis pathway. Therefore, the longitudinal study and randomized controlled trials in humans are essential to determine the role of the gut microbiota in AD and other neurological diseases. Finding potent drugs targeting the microbiome may be more promising for future clinical therapeutic strategies.

## Author Contributions

FZ prepared the manuscript. CL provided views and revised the manuscript. FC and XT helped to correct the manuscript and prepared figures. JZ designed the framework of manuscript, prepared and finalized the manuscript. All authors contributed to the article and approved the submitted version.

## Conflict of Interest

The authors declare that the research was conducted in the absence of any commercial or financial relationships that could be construed as a potential conflict of interest.
